# Comparison of Methods of Clinical Trial Emulation Utilizing Data From the Comparison of AMD Treatment Trial (CATT) and the IRIS® Registry

**DOI:** 10.1016/j.xops.2024.100524

**Published:** 2024-04-03

**Authors:** Helene Fevrier, Andrew LaPrise, Michael Mbagwu, Theodore Leng, Aracelis Z. Torres, Durga S. Borkar

**Affiliations:** 1 Verana Health, San Francisco, California; 2 Byers Eye Institute at Stanford, Stanford University School of Medicine, Palo Alto, California; 3 Duke Eye Center, Duke University School of Medicine, Durham, North Carolina

**Keywords:** Age-related macular degeneration, CATT, Real-world data, Clinical trial emulation, IRIS Registry

## Abstract

**Purpose:**

We used exact matching and inverse propensity score weighting (IPSW) using real-world data (RWD) from the American Academy of Ophthalmology IRIS® Registry (Intelligent Research in Sight) to emulate the 2 pro re nata (*prn*) treatment arms from the Comparison of AMD Treatment Trial (CATT) and to compare the outcomes of the RWD arms to the 2 monthly treatment arms from the clinical trial.

**Design:**

Retrospective cohort study utilizing deidentified electronic health record registry data and patient-level deidentified clinical trial data.

**Subjects:**

All treatment-naive patient eyes with neovascular age-related macular degeneration treated with ranibizumab or bevacizumab only for 1 year from either the CATT or the IRIS Registry.

**Methods:**

Patients were identified in the IRIS Registry between October 1, 2015 and December 31, 2019. After all nonimaging-based inclusion and exclusion criteria from the CATT were applied, patient eyes receiving bevacizumab or ranibizumab only on a *prn* basis were identified as the eligible cohort. Exact matching and ISPW was applied based on age, gender, and baseline visual acuity.

**Main Outcome Measures:**

Mean change in visual acuity, in approximated ETDRS letters, between baseline and 1 year for the IRIS Registry prn treatment arms generated by exact matching and IPSW.

**Results:**

We identified 427 eyes treated with ranibizumab *prn* and 771 eyes treated with bevacizumab *prn*. Using exact matching, 98% (n = 281) of CATT patient eyes in the bevacizumab monthly treatment arm and 87% (n = 261) of CATT patient eyes in the ranibizumab monthly treatment arm were matched to a patient eye in the IRIS Registry. For the ranibizumab *prn* treatment arm, patient eyes generated using exact matching gained 1.9 letters and those generated using IPSW gained 2.8 letters (exact matching: 1.9 letters ± 14.0 vs. IPSW: 2.8 letters ± 15.0 letters, *P* = 0.43). For the bevacizumab *prn* treatment arm, patient eyes generated using exact matching gained 2.4 letters and those generated using IPSW gained 2.1 letters (exact matching: 2.4 letters ± 15.4 vs. IPSW: 2.1 letters ± 16.0 letters, *P* = 0.79).

**Conclusions:**

Both exact matching and IPSW produced similar results in emulating the *prn* treatment arms of the CATT using IRIS Registry data and patient-level clinical trial data. Similar to prior real-world studies, the clinical outcomes were significantly worse in the IRIS Registry treatment arms compared with the clinical trial.

**Financial Disclosure(s):**

Proprietary or commercial disclosure may be found in the Footnotes and Disclosures at the end of this article.

Recent draft guidance from the United States Food and Drug Administration encourages the use of real-world data (RWD), or data generated from routine clinical care, to augment clinical trials in regulatory decision-making.^1^^,^^2^ Real-world data in clinical trials can be used to improve clinical trial recruitment, collect trial data directly from the electronic health record (EHR) at the point of care, ascertain clinical endpoints from administrative claims data, and create real-world control arms from clinical registries.^3^, ^4^, ^5^, ^6^ Although these methods have frequently been used in other medical specialties, such as cardiology and oncology, there has been limited use to streamline the implementation of clinical trials in ophthalmology.^7^

A key application of RWD is constructing standard-of-care or control arms using EHR data collected as part of routine clinical care to compare to an interventional arm in a clinical trial setting.^7^ One known limitation of using RWD to emulate a clinical trial is the inherent differences between treatment selection for real-world patients and clinical trial patients; however, use of advanced statistical methods can help minimize this difference. In order to explore the impact of different methodologies, the Comparison of Age-Related Macular Degeneration Treatment Trials (CATT), a National Eye Institute-funded randomized controlled trial investigating the efficacy of ranibizumab versus bevacizumab treatment for neovascular age-related macular degeneration (nAMD) administered via 2 dosing schedules (monthly and *pro re nata* [prn]), was selected for emulation.^8^ The scale and breadth of the American Academy of Ophthalmology IRIS® Registry (Intelligent Research in Sight), which is the world’s largest ophthalmology EHR registry and contains >6.4 million age-related macular degeneration patients, enabled its use as a source of RWD.

This study aimed to compare 2 methodologies, exact matching and inverse propensity score weighting (IPSW), using RWD from the IRIS Registry to emulate the 2 *prn* treatment arms from CATT and to compare the outcomes of these real-world arms to the 2 monthly treatment arms from the clinical trial.

## Methods

Deidentified data from the IRIS Registry, which, as of July 2022, contained >75 million patients, were used for this analysis. This study complies with the tenets of the Declaration of Helsinki. Data stored within the IRIS Registry were deidentified and have been qualified as Health Insurance Portability and Accountability Act compliant. The study was reviewed and deemed exempt under 45 CFR § 46.104(d)(4) by the WIRB-Copernicus Group Institutional Review Board (Puyallup, WA). No patient consent was required because this study was certified as conducted on anonymized, deidentified data used for research purposes by Verana Health.

All treatment-naive patient eyes with nAMD in the IRIS Registry who received their first anti-VEGF injection between October 1, 2015 and December 31, 2019 were eligible for study inclusion based on *International Classification of Diseases, 10*^*th*^ *Edition* and Current Procedural Terminology coding. Treatment-naive was defined as no documentation of anti-VEGF injections in structured EHR data (Current Procedural Terminology and Healthcare Common Procedure Coding System codes) for 1 year prior to the index date, or date of first injection.

Additional inclusion and exclusion criteria were applied to reflect (1) all nonimaging based eligibility criteria from the CATT and (2) the treatment pattern required in the CATT of *prn* treatment with either bevacizumab or ranbizumab for 1 year.^8^ These are listed in detail in Table 1. Specifically, patient eyes that received bevacizumab only or ranibizumab only for 1 year after the initial index date were included. If a patient was receiving anti-VEGF injections in both eyes during the study period, the eye with the earlier index date was included in the study. As needed or *prn* treatment was defined as a treatment regimen requiring a clinical encounter with a retina specialist every 4 to 6 weeks for 1 year after the index date and ≥1 clinical encounter with a retina specialist that was not associated with an anti-VEGF intravitreal injection.

**Table 1:** Cohort Attrition∗ for IRIS Registry *prn* Bevacizumab and Ranibizumab Cohorts

Selection Criteria	Patients	Patient Eyes	% (Eyes)
Creation of Treatment-Naive nAMD Cohort in the IRIS Registry			
Injection of bevacizumab or ranibizumab in 2015–2019	1 265 993	1 620 330	100.0%
Documentation of nAMD within 1 year preindex	449 643	531 133	32.9%
Age of ≥50 years at the time of index injection	446 609	527 676	32.6%
No anti-VEGF injections received up to 365 days preindex	376 062	435 310	26.9%
≥1 year of preindex data	212 463	242 127	14.9%
Application of CATT Inclusion Criteria and Treatment Regimen Requirements			
Visual acuity between 20/25 and 20/320 in the study eye	125 726	137 113	8.5%
Visit every 4–6 weeks for a period of 365 days (some will be noninjection visits) by a retina specialist	7660	8191	0.5%
Treatment with only bevacizumab or ranibizumab for a period of 365 days	4861	5224	0.3%
Application of CATT Exclusion Criteria			
Patients with diabetic retinopathy or diabetic macular edema in the study eye	4656	5002	0.3%
Vitreous hemorrhage in the study eye	4637	4982	0.3%
History of rhegmatogenous retinal detachment or macular hole in the study eye	4558	4896	0.3%
History of pars plana vitrectomy in the study eye	4553	4888	0.3%
Intraocular surgery (including cataract surgery) in the study eye within 2 months	4515	4846	0.3%
Uncontrolled glaucoma in the study eye (defined as intraocular pressure ≥25 mmHg on ≥2 readings in the year prior to index date)	4470	4795	0.3%
Previous treatment with verteporfin PDT, ranibizumab, intravitreal bevacizumab, in the study eye	4460	4784	0.3%
Fibrosis or geographic atrophy involving the center of the fovea in the study eye (based on subfoveal GA ICD code)	4336	4649	0.3%
CNV in either eye due to other causes, such as ocular histoplasmosis, trauma, or pathologic myopia	4305	4617	0.3%
Patients with other ocular diseases that can compromise the visual acuity of the study eye (amblyopia, anterior ischemic optic neuropathy, pattern dystrophy)	4244	4550	0.3%
Exclude patients receiving anti-VEGF treatment in the fellow eye during the 365 day follow up period	2298	2300	0.1%
Eyes with ≥1 retina specialist visit without an anti-VEGF injection	1198	1198	0.1%

CATT = Comparison of AMD Treatment Trial; CNV = choroidal neovascularization; ICD = International Classification of Diseases; IRIS = Intelligent Research in Sight; nAMD = neovascular age-related macular degeneration; PDT = photodynamic therapy; prn = pro re nata.

∗ Each number under patients and patient eyes represents the cumulative attrition after all criteria in that specific row and all preceding rows have been applied.

Patient demographic data, including age, gender, and race, and clinical information, including visual acuity (VA), number of injections, and incidence of endophthalmitis, was ascertained from IRIS Registry data for the real-world cohorts. Visual acuity was determined based on the best documented Snellen VA of a study eye for the clinical encounter. Best documented Snellen VA was then converted to an approximated ETDRS letter equivalent for statistical analysis using the formula 85 + 50 × log (Snellen fraction) rounded to the nearest letter; this formula has been described in more detail previously.^9^

**Figure 1:**
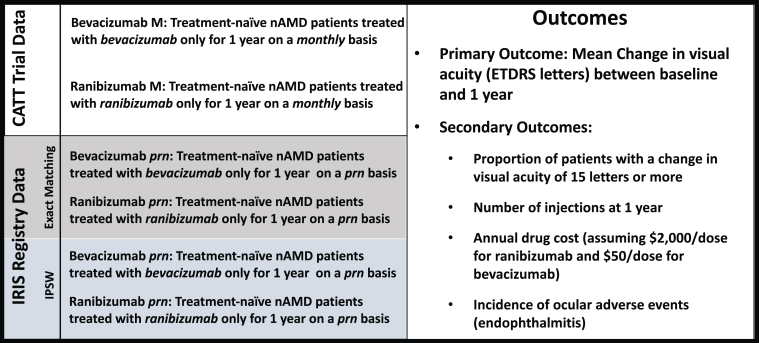
Schematic diagram depicting the study design which compares 2 monthly treatment arms from the CATT trial to bevacizumab and ranibizumab *prn* treatment arms generated using IRIS Registry data and applying both exact matching and inverse propensity score weighting. Primary and secondary outcomes evaluated for each of these treatment arms are described to the right. CATT = Comparison of AMD Treatment Trial; IPSW = inverse propensity score weighting; IRIS = Intelligent Research in Sight; nAMD = neovascular age-related macular degeneration; prn = pro re nata.

After the bevacizumab and ranibizumab cohorts were determined, 2 methodologies were used to generate real-world treatment arms to compare to the monthly ranibizumab only and the monthly bevacizumab only treatment arms from the CATT using deidentified patient-level clinical trial data, which is publicly available at https://hyperprod.cceb.med.upenn.edu/catt/catt_index.php. Figure 1 provides a schematic of the treatment arms generated from IRIS Registry and the outcomes evaluated. Exact matching was used to match a patient eye 1:1 from the IRIS Registry to a patient eye in the CATT on the basis of age, gender, and baseline VA at the time of initial anti-VEGF treatment, similar to what was done in a prior United Kingdom-based paper utilizing exact matching to create a synthetic control arm for an nAMD clinical trial.^7^ Age was matched within 5 years of the clinical trial participant age at baseline visit, with the exception of patients ≥90 years of age, who were matched into this broader category due to deidentification purposes. Visual acuity was matched within 5 letters of baseline VA between CATT patients and IRIS Registry patients.

Inverse propensity score weighting was also used to generate a real-world *prn* cohort for bevacizumab and ranibizumab. A logistic regression model was used to predict probability of exposure to either bevacizumab or ranibizumab and either *prn* or monthly treatment based on age, gender, and baseline VA. These results were then used to weight observations in the IRIS Registry *prn* bevacizumab and ranibizumab treatment arms.

The main nonimaging based primary and secondary outcomes were evaluated for the real-world cohorts. Specifically, the primary outcome that was evaluated for the IRIS Registry *prn* treatment arms was the mean change in VA, in approximated ETDRS letters, between baseline and 1 year. The secondary outcomes evaluated were proportion of patient eyes with a change in VA of ≥15 letters at 1 year, number of injections at 1 year, annual drug cost (assuming $2000 per dose for ranibizumab and $50 per dose for bevacizumab), and incidence of ocular adverse events.

Clinical outcomes were compared between the exact matching and IPSW *prn* cohorts for bevacizumab and ranibizumab respectively. Additionally the *prn* cohorts were compared with the respective monthly cohorts from the CATT. All analyses were conducted using Python 3.7 and Apache Spark 2.4.5 (Apache Software Foundation). *P*-values were calculated using an unpaired t-test, and a *P*-value <0.05 was considered statistically significant.

## Results

Overall, 435 310 eyes of 376 062 patients with nAMD who initiated anti-VEGF treatment with ranibizumab or bevacizumab were identified during the study period. After nonimaging-based inclusion and exclusion criteria, and required treatment regimen criteria were applied, 427 eyes treated with ranibizumab *prn* and 771 eyes treated with bevacizumab *prn* were identified. The details of cohort attrition are provided in Table 1. Baseline characteristics, including age, gender, race, and baseline VA, which are described in Table 2, were comparable between the CATT and eligible IRIS Registry patients.

**Table 2:** Baseline Characteristics for CATT Monthly Treatment Arms and IRIS Registry *prn* Bevacizumab and Ranibizumab RWD Cohorts

Demographic	CATT Trial Data				IRIS Overall Real-World Cohorts			
	Ranibizumab Monthly (N = 301)		Bevacizumab Monthly (N = 286)		Ranibizumab *prn* (N = 427)		Bevacizumab *prn* (N = 771)	
	n (or Mean)	% (or 95% CI)	n (or Mean)	% (or 95% CI)	n (or Mean)	% (or 95% CI)	n (or Mean)	% (or 95% CI)
Age mean, SD	79.16	(64.97, 93.35)	79.90	(66.20, 93.60)	81.16	(66.56, 95.76)	81.05	(65.43, 96.67)
Sex								
Female	183	60.8%	180	62.9%	293	68.9%	474	61.8%
Male	118	39.2%	106	37.1%	132	31.1%	293	38.2%
Race								
White	297	98.7%	281	98.3%	388	90.9%	696	90.3%
Other	4	1.3%	5	1.8%	10	2.3%	25	3.2%
Unknown	0	0%	0	0%	29	6.8%	50	6.5%
Visual acuity score and Snellen equivalent								
68–82 letters, 20/25–20/40	111	36.9%	94	32.9%	150	35.1%	265	34.4%
53–67 letters, 20/50–20/80	98	32.6%	118	41.3%	178	41.7%	318	41.3%
38–52 letters, 20/100–20/160	67	22.3%	53	18.5%	44	10.3%	108	14%
23–37 letters, 20/200–20/320	25	8.3%	21	7.3%	55	12.9%	80	10.4%
Mean number of letters, SD	60.11	14.26	60.16	13.12	60.28	13.22	60.15	12.48

CATT = Comparison of AMD Treatment Trial; CI = confidence interval; IRIS = Intelligent Research in Sight; M = monthly; nAMD = neovascular age-related macular degeneration; prn = pro re nata; RWD = real-world data; SD = standard deviation.

After exact matching, the CATT bevacizumab monthly treatment arm was comprised of 286 patient eyes, and the ranibizumab monthly treatment arm was comprised of 301 patient eyes.^8^ When matched on the basis of age, gender, and baseline VA, 98% (n = 281) of CATT patient eyes in the bevacizumab monthly treatment arm and 87% (n = 261) of CATT patient eyes in the ranibizumab monthly treatment arm were able to be exact matched to a patient eye in the IRIS Registry, respectively.

Inverse propensity score weighting was then performed on the basis of age, gender, and baseline VA, to provide weights for each of the patient eyes in the IRIS Registry that were identified to meet the clinical trial eligibility criteria and received bevacizumab *prn* (n = 771) or ranibizumab *prn* (n = 427) for 1 year. Baseline characteristics for the exact matching and IPSW arms, including age, gender, race, and baseline VA, are described in Table 3.

**Table 3:** Baseline Characteristics for *prn* Cohorts Created by Exact Matching and Inverse Propensity Score Weighting

Demographic	Exact Matching				Inverse Propensity Score Weighting			
	Ranibizumab *prn* (N = 261)		Bevacizumab *prn* (N = 281)		Ranibizumab *prn* (N = 427)		Bevacizumab *prn* (N = 771)	
	n (or Mean)	% (or 95% CI)	n (or Mean)	% (or 95% CI)	n (or Mean)	% (or 95% CI)	n (or Mean)	% (or 95% CI)
Age year	79.75	(66.23, 93.27)	80.04	(66.34, 93.74)	80.29	(67.69, 92.89)	80.71	(70.71, 90.71)
Sex								
Female	160	61.3%	180	64.1%	279.4	65.6%	476.8	62.1%
Male	101	38.7%	101	35.9%	146.4	34.4%	291.3	37.9%
Race								
White	234	89.7%	253	90%	386.0	90.4%	694.3	90.1%
Other	8	3.1%	11	3.9%	10.2	2.4%	26.4	3.4%
Unknown	19	7.3%	17	6.1%	30.9	7.2%	50.3	6.5%
Visual acuity score and Snellen equivalent								
68–82 letters, 20/25–20/40	104	39.9%	95	33.8%	150.5	40.7%	265.7	38.5%
53–67 letters, 20/50−20/80	96	36.8%	115	40.9%	174.7	47.3%	317.3	45.9%
38–52 letters, 20/100−20/160	38	14.6%	52	18.5%	44.2	12%	107.9	15.6%
23–37 letters, 20/200−20/320	23	8.8%	19	6.8%	0.0	0%	0.0	0%
Mean number of letters	61.14	12.82	60.36	12.29	60.10	15.41	60.15	12.92

CI = confidence interval; prn = pro re nata; SD = standard deviation.

### PRN vs. Monthly Treatment Arms

Clinical outcomes were then evaluated and are described in Table 4 for the CATT monthly and *prn* ranibizumab and bevacizumab treatment arms and for the IRIS Registry *prn* treatment arms generated by exact matching and IPSW. Overall, fewer intravitreal injections were performed in the *prn* treatment arms from the IRIS Registry compared with the monthly treatment arms from the CATT for both bevacizumab and ranibizumab. Additionally, VA outcomes were significantly worse in the *prn* treatment arms from the IRIS Registry compared with the monthly treatment arms from the CATT for both bevacizumab and ranibizumab. The VA changes for the CATT treatment arms and the VA changes for the IRIS Registry cohort for both exact matching and IPSW are presented in Table 4. All *P*-values comparing CATT monthly VA outcomes with IRIS *prn* VA outcomes for IPSW and exact matching were 0.001 for both ranibizumab and bevacizumab.

**Table 4:** Clinical Outcomes for CATT Patients in *prn* and Monthly Cohorts and IRIS Registry Simulated *prn* Arms^4^

Outcome	CATT Trial Data								IRIS Registry: Exact Matching				IRIS Registry: Inverse Propensity Score Weighting			
	Ranibizumab M (N = 284)		Bevacizumab M (N = 265)		Ranibizumab *prn* (N = 285)		Bevacizumab *prn* (N = 271)		Ranibizumab *prn* (N = 261)		Bevacizumab *prn* (N = 281)		Ranibizumab *prn* (N = 427)		Bevacizumab *prn* (N = 771)	
	n (or Mean)	% (or 95% CI)	n (or Mean)	% (or 95% CI)	n (or Mean)	% (or 95% CI)	n (or Mean)	% (or 95% CI)	n (or Mean)	% (or 95% CI)	n (or Mean)	% (or 95% CI)	n (or Mean)	% (or 95% CI)	n (or Mean)	% (or 95% CI)
Change from baseline visual acuity score																
Letters: Mean change	8.5	(−19.14, 36.14)	8	(−22.97, 38.97)	6.8	(−18.88, 32.48)	5.9	(−24.87, 36.67)	1.9	(−25.50, 29.30)	2.4	(−27.71, 32.51)	2.8	(−26.70, 32.30)	2.1	(−29.20, 33.40)
Increase of ≥15 letters	97	34.20%	83	31.30%	71	24.90%	76	28.00%	42	17.60%	49	18.70%	74.5	19.10%	131	18.80%
Decrease of ≥15 letters	16	5.60%	16	6.00%	13	4.60%	23	8.50%	29	12.10%	24	9.20%	41.6	10.70%	81.1	11.60%
Treatments																
Mean number of injections	11.7	(8.76, 14.64)	11.9	(9.55, 14.25)	6.9	(1.02, 12.78)	7.7	(0.84, 14.56)	9	(2.34, 15.66)	7.9	(1.04, 14.76)	9	(1.55, 16.45)	7.7	(0.64, 14.76)
Cost Analysis																
Average cost of drug/patient	$23 400		$595		$13 800		$385		$18 060.00		$396.50		$18 060.00		$382.50	
Adverse Events																
Endophthalmitis	2	0.70%	4	1.40%	0	0.00%	0	0.00%	0	0.00%	0		0		0	

CATT = Comparison of AMD Treatment Trial; CI = confidence interval; IRIS = Intelligent Research in Sight; M = monthly, *prn* = pro re nata; SD = standard deviation.

### Exact Matching vs. IPSW

Both exact matching and IPSW yielded very similar results in terms of change in VA at 1 year, as well as proportion of patient eyes with a change in VA of ≥15 letters at 1 year, number of injections at 1 year, annual drug cost, and incidence of endophthalmitis. Specifically, patient eyes in the ranibizumab *prn* treatment arm generated using exact matching gained 1.9 letters and patient eyes in the ranibizumab *prn* treatment arm generated using IPSW gained 2.8 letters (exact matching: 1.9 letters ± 14.0 vs. IPSW: 2.8 letters ± 15.0 letters, *P* = 0.43). Similarly, patient eyes in the bevacizumab *prn* treatment arm generated using exact matching gained 2.4 letters and patient eyes in the bevacizumab *prn* treatment arm generated using IPSW gained 2.1 letters (exact matching: 2.4 letters ± 15.4 vs. IPSW: 2.1 letters ± 16.0 letters, *P* = 0.79). The number of injections in the ranibizumab *prn* treatment arms generated using the 2 methodologies was exactly the same, 9.0 injections, and the number of injections in the bevacizumab *prn* treatment arms generated using exact matching and IPSW was 7.9 and 7.7 injections, respectively.

## Discussion

Prior studies evaluating the role of RWD to emulate clinical trial arms in ophthalmology have been limited.^7^^,^^10^ The purpose of this study was to compare 2 methodologies, exact matching and IPSW, using RWD from the IRIS Registry to emulate the 2 *prn* treatment arms from the CATT and to compare the outcomes of these real-world arms to the 2 monthly treatment arms from the clinical trial. Our study used approximated ETDRS letters as its primary clinical outcome and also evaluated injection frequency as a secondary outcome.

This study demonstrated that the 2 methodologies, exact matching and IPSW, generated treatment arms with very similar clinical outcomes, including change in VA at 1 year and number of injections in 1 year. Additionally, the *prn* treatment arms from the IRIS Registry had worse outcomes with respect to VA gains compared with the monthly treatment arms from the CATT.

There are multiple possible reasons why the 2 methodologies produced such similar results. For one, both the inclusion and exclusion criteria and the treatment regimen requirements were so specific that they yielded a cohort of patient eyes that were very similar due to adjustments made by restriction. Of the 1.6 million treatment-naive patient eyes treated with bevacizumab or ranibizumab in the IRIS Registry, <0.1% met all the nonimaging-based inclusion and exclusion criteria and followed the appropriate treatment regimen. This subgroup of patient eyes was comparable in age, gender, and baseline VA to the CATT monthly treatment arms, suggesting that IPSW may not have adjusted this overall cohort significantly. Because the overall cohort was small, over one-half of the ranibizumab-treated eyes and more than one-third of bevacizumab-treated eyes were included in the exact matching cohort and may partially explain why similar results were observed. Additionally, the scale of the IRIS Registry allowed for the use of exact matching. Other methodologies may be required for instances where the size of a source population for a study is limited.

This study also demonstrated that clinical outcomes in the IRIS Registry *prn* treatment arms were significantly worse than the treatment outcomes in the CATT monthly treatment outcomes. Specifically, patient eyes in the CATT monthly treatment arms for ranibizumab and bevacizumab gained 8.5 and 8.0 letters respectively at 1 year compared with between 2 and 3 letters gained in the IRIS Registry *prn* treatment arms. Rather than suggest that *prn* treatment is inferior to monthly treatment, which would be contrary to the CATT results,^8^ this validates the results of several prior RWD studies showing that real-world patient outcomes are significantly worse than clinical trial patient outcomes.^11^, ^12^, ^13^, ^14^, ^15^, ^16^ *Pro re nata* treatment in the real-world is likely disrupted by issues, such as insurance coverage and inadvertent lapses in follow up, that are not especially problematic in clinical trials. This is potentially due to other unmeasured or unobserved factors that resulted in continued differences between the trial participants and the RWD cohorts.

Notably, outcomes for patient eyes in the IRIS Registry *prn* treatment arms were worse than the respective *prn* treatment arms in the CATT. The patients in the IRIS Registry ranibizumab *prn* treatment arm in particular who were being seen monthly and treated *prn* were injected more frequently than the *prn* treatment arm in the actual CATT trial. This suggests they may have been more refractory to treatment and is also reflected in the worse VA gains seen in the IRIS Registry *prn* treatment arms despite the same or more frequent treatment. Based on these study results, future trial emulation studies may be better suited to clinical trials that do not have a *prn* treatment arm since true *prn* treatment is difficult to ascertain from structured EHR data alone.

There are limited examples of similar studies in the retina literature.^7^ One prior study in the United Kingdom created a synthetic aflibercept treatment arm using data from the United Kingdom Age-Related Macular Degeneration EMR Users Group to compare to the bevacizumab clinical trial treatment arm for the ABC (Bevacizumab for neovascular age-related macular degeneration) trial.^7^^,^^17^ This study also used exact matching and IPSW considering age, gender, and baseline VA and included only patient eyes receiving standard-of-care treatment. Similar results were noted in this prior study. Specifically, IPSW and exact matching yielded equivocal results in generating a synthetic aflibercept control arm.

There are several strengths to this study. The IRIS Registry is the world’s largest single specialty EHR Registry with >6 million nAMD patients.^18^ This allowed very high matching rates for the bevacizumab and ranibizumab *prn* treatment arms despite very specific inclusion and exclusion criteria, as well as less commonly used treatment regimens during the study period. Additionally, this proof-of-concept study illustrated a novel application for integrating RWD and clinical trial data using deidentified patient-level clinical trial data, which is unique in ophthalmology. Furthermore, the focus on clinical outcomes and adverse events that are routinely captured in the EHR, such as VA, number of intravitreal injections, and endophthalmitis, allowed for an evaluation of several of the clinical trial outcomes using the simulated *prn* treatment arms.

Despite the strengths of this novel methodology study, there are some limitations to consider. Because data from an EHR registry were used, clinical variables such as VA were not measured in a standardized fashion. In this study, VA was obtained within the IRIS Registry by extracting Snellen VA chart measurements sourced from the practice’s EHR. Although these measurements are less standardized than those in a clinical trial, they are an accurate reflection of real-world clinical practice. Imaging data were not included and as a result, several anatomical inclusion and exclusion criteria in the CATT such as OCT and fluorescein angiography findings, as well as anatomical outcomes, could not be assessed.^8^

Furthermore, while we used a structured approach to define *prn* and monthly treatment, we did not review clinical notes to confirm treatment regimen. Thus, it is possible that not all patients in the *prn* arm were intentionally being treated in a *prn* manner. Additionally, this study used age, gender, and baseline VA to perform exact matching and IPSW. There are several other relevant characteristics including insurance status, individual physician and practice preferences, and social determinants of health that can impact both agent choice and frequency of visits and treatment.^19^, ^20^, ^21^ A study that takes into account some of these factors could be a consideration for future work.

While there are limitations to acknowledge, the applications of this methodology are vast. The CATT was chosen for this study due to the large number of nAMD patients receiving anti-VEGF treatment for this condition, as well as the ability to access publicly-available deidentified patient-level clinical trial data. Both were necessary to apply these methodologies. In the future, there is an opportunity to apply these same methods to more recent clinical trials and across other disease states in ophthalmology, as well as the chance to integrate this methodology prospectively in future clinical trials.

The value of these methodologies could lie in the ability to expedite evaluation of therapeutic agents in a clinical trial setting for disease states that are less common or currently have no treatment if data used in clinical care were used to generate a synthetic control arm. For example, clinical trials in uncommon inherited retinal degenerations or neuro-ophthalmic conditions could potentially be streamlined significantly with the use of real-world control arms. Focusing on disease states that meet the qualifications of having no commercially available treatment could also further minimize the limitation noted in this study that nonrandom factors impact treatment choice and regimen in the real-world. The results of this paper could help stimulate discussion for future novel applications of real-world data to generate synthetic control arms in ophthalmology clinical trials.

## References

[1] (2023). https://www.fda.gov/regulatory-information/search-fda-guidance-documents/considerations-design-and-conduct-externally-controlled-trials-drug-and-biological-products.

[2] (2021). https://www.fda.gov/regulatory-information/search-fda-guidance-documents/real-world-data-assessing-electronic-health-records-and-medical-claims-data-support-regulatory.

[3] Tan K., Bryan J., Segal B. (2022). Emulating control arms for cancer clinical trials using external cohorts created from electronic health record-derived real-world data. Clin Pharmacol Ther.

[4] Brennan J.M., Wruck L., Pencina M.J. (2019). Claims-based cardiovascular outcome identification for clinical research: results from 7 large randomized cardiovascular clinical trials. Am Heart J.

[5] Popat S., Liu S.V., Scheuer N. (2022). Addressing challenges with real-world synthetic control arms to demonstrate the comparative effectiveness of pralsetinib in non-small cell lung cancer. Nat Commun.

[6] Evans S.R., Paraoan D., Perlmutter J. (2021). Real-world data for planning eligibility criteria and enhancing recruitment: recommendations from the clinical trials transformation initiative. Ther Innov Regul Sci.

[7] Thomas D.S., Lee A.Y., Muller P.L. (2021). Contextualizing single-arm trials with real-world data: an emulated target trial comparing therapies for neovascular age-related macular degeneration. Clin Transl Sci.

[8] Group C.R., Martin D.F., Maguire M.G. (2011). Ranibizumab and bevacizumab for neovascular age-related macular degeneration. N Engl J Med.

[9] Gregori N.Z., Feuer W., Rosenfeld P.J. (2010). Novel method for analyzing Snellen visual acuity measurements. Retina.

[10] Vanner E.A., Sun C.Q., McSoley M.J. (2021). The Tube Versus Trabeculectomy IRIS(R) Registry Study: cohort selection and follow-up and comparisons to the randomized controlled trial. Am J Ophthalmol.

[11] Rao P., Lum F., Wood K. (2018). Real-world vision in age-related macular degeneration patients treated with single anti-VEGF drug type for 1 year in the IRIS Registry. Ophthalmology.

[12] Ciulla T.A., Hussain R.M., Taraborelli D. (2022). Longer-term anti-VEGF therapy outcomes in neovascular age-related macular degeneration, diabetic macular edema, and vein occlusion-related macular edema: clinical outcomes in 130 247 eyes. Ophthalmol Retina.

[13] Mehta H., Kim L.N., Mathis T. (2020). Trends in real-world neovascular AMD treatment outcomes in the UK. Clin Ophthalmol.

[14] Kiss S., Campbell J., Almony A. (2020). Management and outcomes for neovascular age-related macular degeneration: analysis of United States electronic health records. Ophthalmology.

[15] Khanani A.M., Skelly A., Bezlyak V. (2020). SIERRA-AMD: a retrospective, real-world evidence study of patients with neovascular age-related macular degeneration in the United States. Ophthalmol Retina.

[16] Verbraak F.D., Ponsioen D.L., Tigchelaar-Besling O.A.M. (2021). Real-world treatment outcomes of neovascular age-related macular degeneration in the Netherlands. Acta Ophthalmol.

[17] Patel P.J., Bunce C., Tufail A., Investigators A.B.C.T. (2008). A randomised, double-masked phase III/IV study of the efficacy and safety of Avastin(R) (Bevacizumab) intravitreal injections compared to standard therapy in subjects with choroidal neovascularisation secondary to age-related macular degeneration: clinical trial design. Trials.

[18] Lee C.S., Blazes M., Lorch A. (2022). American Academy of Ophthalmology Intelligent Research in Sight (IRIS(R)) Registry and the IRIS Registry Analytic Center Consortium. Ophthalmol Sci.

[19] Obeid A., Gao X., Ali F.S. (2018). Loss to follow-up among patients with neovascular age-related macular degeneration who received intravitreal anti-vascular endothelial growth factor injections. JAMA Ophthalmol.

[20] Soares R.R., Mellen P., Garrigan H. (2020). Outcomes of eyes Lost to follow-up with neovascular age-related macular degeneration receiving intravitreal anti-vascular endothelial growth factor. Ophthalmol Retina.

[21] Parikh R., Ross J.S., Sangaralingham L.R. (2017). Trends of anti-vascular endothelial growth factor use in ophthalmology among privately insured and medicare advantage patients. Ophthalmology.

